# Term Human Placental Trophoblasts Express SARS-CoV-2 Entry Factors ACE2, TMPRSS2, and Furin

**DOI:** 10.1128/mSphere.00250-21

**Published:** 2021-04-14

**Authors:** Yingshi Ouyang, Tarique Bagalkot, Wendy Fitzgerald, Elena Sadovsky, Tianjiao Chu, Ana Martínez-Marchal, Miguel Brieño-Enríquez, Emily J. Su, Leonid Margolis, Alexander Sorkin, Yoel Sadovsky

**Affiliations:** a Department of Obstetrics, Gynecology and Reproductive Sciences, Magee-Womens Research Institute, University of Pittsburgh School of Medicine, Pittsburgh, Pennsylvania, USA; b Department of Cell Biology, University of Pittsburgh School of Medicine, Pittsburgh, Pennsylvania, USA; c Section on Intercellular Interaction, Eunice Kennedy Shriver National Institute of Child Health and Human Development, National Institutes of Health, Bethesda, Maryland, USA; d Department of Obstetrics and Gynecology, University of Colorado School of Medicine, Aurora, Colorado, USA; e Department of Microbiology and Molecular Genetics, University of Pittsburgh School of Medicine, Pittsburgh, Pennsylvania, USA; University of Maryland School of Medicine

**Keywords:** placenta, trophoblast, SARS-CoV-2, ACE2, TMPRSS2, furin

## Abstract

The coronavirus disease 2019 (COVID-19) pandemic, caused by severe acute respiratory syndrome coronavirus 2 (SARS-CoV-2), has had a massive impact on human lives worldwide. While the airborne SARS-CoV-2 primarily affects the lungs, viremia is not uncommon. As placental trophoblasts are directly bathed in maternal blood, they are vulnerable to SARS-CoV-2. Intriguingly, the human fetus is largely spared from SARS-CoV-2 infection. We tested whether the human placenta expresses the main SARS-CoV-2 entry factors angiotensin-converting enzyme 2 (ACE2), transmembrane protease serine 2 (TMPRSS2), and furin and showed that ACE2 and TMPRSS2 are expressed in the trophoblast rather than in other placental villous cells. While furin is expressed in the main placental villous cell types, we surveyed, trophoblasts exhibit the highest expression. In line with the expression of these entry factors, we demonstrated that a SARS-CoV-2 pseudovirus could enter primary human trophoblasts. Mechanisms underlying placental defense against SARS-CoV-2 infection likely involve postentry processing, which may be germane for mitigating interventions against SARS-CoV-2.

**IMPORTANCE** Pregnant women worldwide have been affected by COVID-19. As the virus is commonly spread to various organs via the bloodstream and because human placental trophoblasts are directly bathed in maternal blood, feto-placental infection by SARS-CoV-2 seems likely. However, despite the heightened risk to pregnant women, thus far the transmission risk of COVID-19 to the feto-placental unit seems extremely low. This has been recently attributed to a negligible expression of SARS-CoV-2 entry factors in the human placenta. We therefore sought to explore the expression of the entry factors ACE2 and TMPRSS2 in the different cell types of human placental villi. Using a combination of transcriptome sequencing (RNA-seq), real-time quantitative PCR (RT-qPCR), *in situ* hybridization, and immunofluorescence, we found that trophoblasts, but not the other main villous cell types, express ACE2 and TMPRSS2, with a broad expression of furin. Correspondingly, we also showed that primary human trophoblasts are permissive to entry of SARS-CoV-2 pseudovirus particles.

## INTRODUCTION

The severe acute respiratory syndrome coronavirus 2 (SARS-CoV-2), a positive-sense, single-stranded RNA virus, is responsible for the coronavirus disease 2019 (COVID-19) pandemic that continues to affect millions of human lives worldwide. There is a pressing and unmet need to understand the pathogenesis of SARS-CoV-2, promote disease prevention, enhance diagnostics, repurpose existing therapeutics and innovate new ones, and provide safe pipelines for the development of effective vaccines. Whereas the pathogenesis of COVID-19 remains incompletely understood, accumulating evidence indicates that the initial viral breaching of the immune defense, followed by robust viral replication in the lungs (1), may lead to a systemic cytokine storm (2) that can ultimately result in multiorgan damage or failure.

Angiotensin-converting enzyme 2 (ACE2) was initially identified as a cell membrane carboxypeptidase that modulates blood pressure, including blood pressure during pregnancy, by producing the vasodilator heptapeptide angiotensin-(1–7) (3–5). The viral spike (S) protein of SARS-CoV-2 uses ACE2 receptors as its binding receptor on the host cell membrane (6), thus enabling the subsequent entry of the virus into host cells after proteolytic cleavage of the S protein by transmembrane protease serine 2 (TMPRSS2). This leads to exposure of a fusogenic peptide that promotes fusion of the viral envelope with the host cell membrane and the ensuing endocytosis and viral replication (1, 7). Notably, unlike SARS-CoV, SARS-CoV-2 appears to require an additional cleavage at the S1/S2 site of the S protein by the proprotein convertase furin to enable viral assembly in host cells and to mediate viral spread to permissive cells (8, 9).

Pregnancy poses an increased risk to certain viral infections, such as cytomegalovirus (CMV), rubella, and Zika virus, leading to major fetal morbidity and mortality (10, 11). Due to the high infectivity of SARS-CoV-2 and its transmission via respiratory secretions or droplets, the transmission of SARS-CoV-2 to pregnant women is inevitable. According to the CDC’s report of a COVID-19-associated hospitalization surveillance network across 13 states until 22 August 2020, approximately 45.5% of pregnant women confirmed as having COVID-19 manifested symptoms leading to admission to an intensive care unit (16.2%) and/or the need for mechanical ventilation (8.5%) (12). The risk is heightened in mothers with preexisting morbid conditions (13, 14). In light of the physiological reduction in lung volume that characterizes pregnancy and based on clinical experience from influenza, SARS-CoV-1, H1N1, and Middle East respiratory syndrome (MERS)-CoV infections, severe respiratory infections may worsen during pregnancy, thus potentially exacerbating maternal morbidity and mortality (15–19). Several reports point to a higher incidence of preterm delivery, premature rupture of the membranes, and other pregnancy complications in SARS-CoV-2-infected mothers (20–22). Notably, these obstetrical complications have been associated with infection by SARS-CoV-1, MERS-CoV, metapneumovirus, respiratory syncytial virus, and influenza virus and largely attributed to maternal respiratory complications and consequent hypoxemia (23–25).

Because (i) human placental trophoblasts are directly bathed in maternal blood and (ii) a high fraction of the cardiac output reaches the human placenta, hematogenous spread to the placenta by blood-borne viruses is likely. Some airborne viruses, such as rubella virus, may cause viremia and affect the feto-placental unit. Similarly, the presence of SARS-CoV-2 viral RNA in the blood (RNAemia) has been detected in 8 to 40% of hospitalized COVID-19 patients (26–29), which may explain the hematogenous spread of SARS-CoV-2 to numerous internal organs (30–33). Thus, trophoblasts at the maternal-fetal interface may directly confront circulating SARS-CoV-2 particles in the blood of COVID-19-positive pregnant women.

Unlike rubella virus, CMV, and Zika virus, most data thus far suggest that the incidence of feto-placental transmission of SARS-CoV-2 is very low, even in mothers with severe clinical COVID-19 (34–41). Although there have been reports of SARS-CoV-2 detection in neonatal nasopharyngeal swabs, amniotic fluid, or cord blood, with elevated immunoglobulin M (IgM) levels in the neonatal blood, these have been rare. Some of these assays were followed by a negative test for viral RNA in the neonate (42, 43). In addition, the reliability of the results has been questioned, mainly due to uncertainty regarding the specificity of the IgM assay (35, 44). Similarly, the diagnostic value of detecting SARS-CoV-2 RNA by qPCR in placental biopsy specimens (39–41, 45–47) is questionable in light of the uncertainty regarding contamination with maternal blood.

The potential ability of SARS-CoV-2 to infect human placental trophoblasts depends on the presence of receptors and processing proteins that mediate trophoblast viral entry. Thus far, such data have been scant and relatively inconsistent. SARS-CoV-2 viral proteins, including S and N (nucleocapsid) proteins, were visualized in syncytiotrophoblasts, which are in direct contact with the maternal circulation (45–48). However, a recent analysis of single-cell transcriptome sequencing (RNA-seq) suggested negligible ACE2 and TMPRSS2 expression in the placenta (49). In contrast, earlier studies showed that ACE2 is expressed in the human placenta, especially in syncytiotrophoblasts, the subjacent cytotrophoblasts, and endothelial cells of the umbilical cord (5, 45, 50–58). The expression of placental ACE2 is increased in patients with preeclampsia, suggesting the functional role of ACE2 in countering hypertension at the human uteroplacental interface (5, 50). Less information exists regarding ACE2 expression in the various villous cell types and the expression of TMPRSS2 and furin. We therefore sought to explore the expression of ACE2 and TMPRSS2 in the different cell types of placental villi. The expression of these processing proteins would suggest that trophoblasts are susceptible to SARS-CoV-2 infection and the ensuing transmission to the fetus. Using a combination of RNA-seq, real-time quantitative PCR (RT-qPCR), *in situ* hybridization-based RNAscope, and immunofluorescence, we found that trophoblasts, but not the other main placental villous cell types, express ACE2 and TMPRSS2 with broad expression of furin. Correspondingly, we also showed that primary human trophoblasts derived from term placentas are permissive to entry of SARS-CoV-2 pseudovirus particles.

## RESULTS

### The expression of *ACE2*, *TMPRSS2*, and *furin* mRNA in the human placenta and in villous cells.

**FIG 1:**
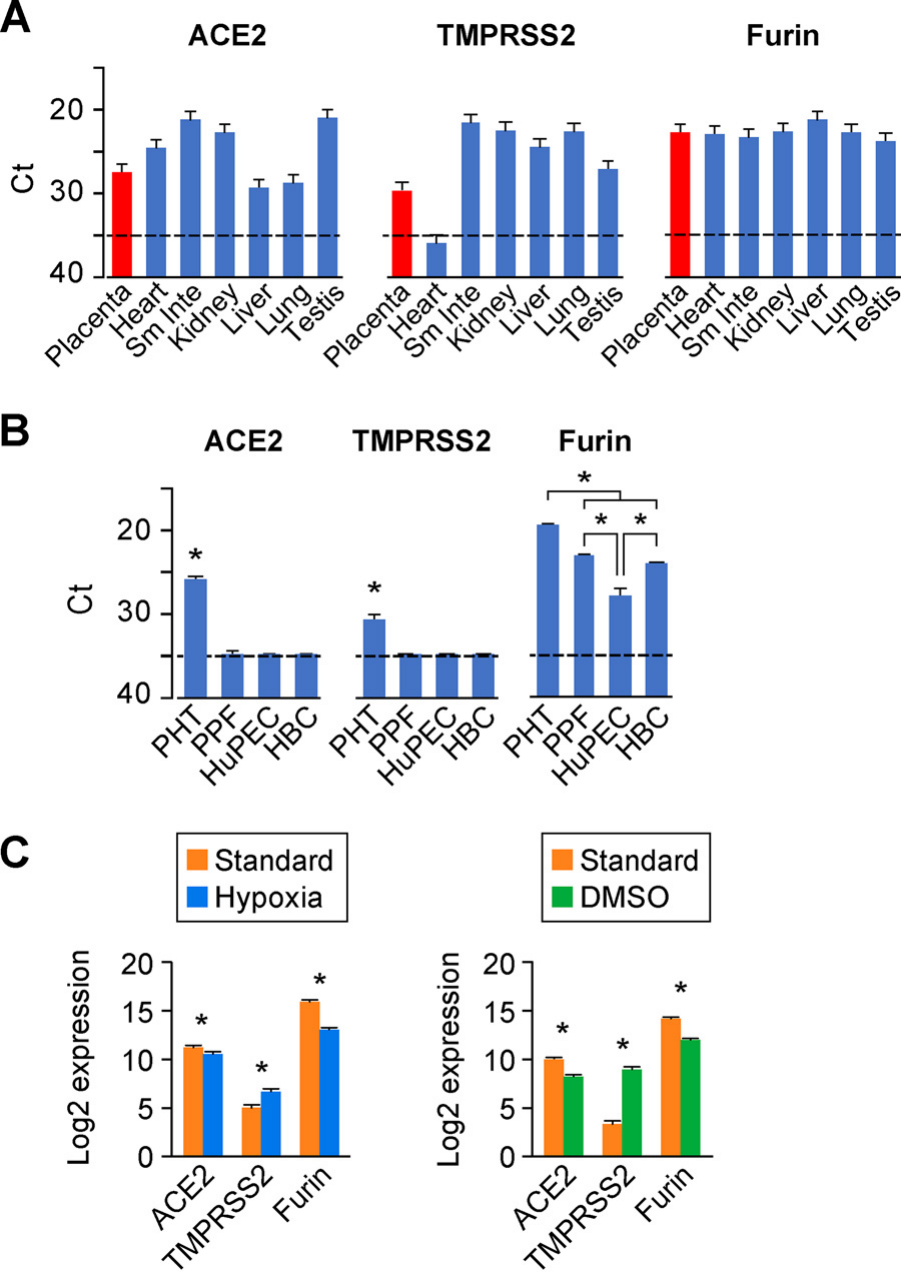
The expression landscape of *ACE2*, *TMPRSS2*, and *furin* in the human placenta and placental cells. (A) The expression of the processing proteins in human organs (Sm Inte, small intestine). Transcripts of ACE2, TMPRSS2, and furin were detected by RT-qPCR using the FirstChoice Human Total RNA Survey Panel. Expression levels are shown by threshold cycle (Ct) values in various human organs. *n* = 3. (B) The expression of the processing proteins in primary human placental villous cells. Data are derived from cultures of the major primary human placenta cells, including trophoblasts (PHT), fibroblasts (PPF), endothelial cells (HuPEC), and Hofbauer cells (HBC). Expression (mRNA), detected by RT-qPCR, is shown by Ct values. *n* = 3. *, *P* < 0.01, ANOVA with *post hoc* Bonferroni test. For panels A and B, the dashed line represents the assay threshold for the presence of each transcript. Note that lower Ct values indicate higher expression. (C) The expression of the processing proteins in cytotrophoblasts versus syncytiotrophoblasts. Standard/hypoxic conditions (left) and culture in the presence of 1.5% DMSO (right) were performed and analyzed as described in Materials and Methods. RNA-seq data (*n* = 5) are expressed as log_2_ value. *, *P* < 0.01.

We first used RT-qPCR as a sensitive tool to quantify the expression of three viral entry factors, *ACE2*, *TMPRSS2*, and *furin*, in term human placenta and compared these data to the expression of these transcripts in other human organs, including the heart, small intestine, kidney, liver, lung, and testis. As shown in Fig. 1A, *ACE2*, *TMPRSS2*, and *furin* were clearly expressed in the human placenta, with average threshold cycle (*C_T_*) values of 27.5, 29.9, and 22.7, respectively. Intriguingly, the expression level of *ACE2* mRNA in the placenta was similar to its expression in the lung, the most common site for SARS-CoV-2 infection in humans. *TMPRSS2* levels in most tissues were higher than the placenta, and the expression of *furin* was fairly comparable among the tissues (Fig. 1A).

To further determine the placental villous cell types that express mRNA of *ACE2*, *TMPRSS2*, and *furin*, we used RT-qPCR to measure these transcripts in the four main villous cell types: primary human trophoblasts (PHT cells), primary placental fibroblasts (PPF cells), primary human placental microvascular endothelial cells (HuPECs), and placental macrophage Hofbauer cells (HBCs). Notably, we routinely culture PHT cells up to 72 h, which has been rigorously tested to ensure their differentiation to the fused multinucleated syncytiotrophoblast phenotype. Interestingly, *ACE2* was clearly expressed in PHT cells, whereas expression in PPF, HuPEC, and HBC cells was below the PCR detection threshold. Likewise, *TMPRSS2* was also discernibly expressed in PHT cells, but not in the other three cell types. All four main placental cell types expressed furin, with PHT cells exhibiting the highest expression levels (Fig. 1B). These results indicate that, among the main placental villous cell types, only trophoblasts express transcripts for the three key SARS-CoV-2 entry factors. Our data also indicate that furin is ubiquitously expressed in all major cell types of human placenta villi.

We used bulk RNA-seq to validate the expression of *ACE2*, *TMPRSS2*, and *furin* in trophoblasts (Fig. 1C). To profile their expression levels in differentiated syncytiotrophoblasts versus progenitor cytotrophoblasts, we cultured PHT cells in two conditions that are known to hinder the differentiation of cytotrophoblasts to syncytiotrophoblasts *in vitro*, namely, culture in the presence of 1.5% dimethyl sulfoxide (DMSO) or in hypoxia (<1% O_2_ for 48 h) (59–62). We found that both conditions resulted in a moderate, yet statistically significant, decrease of *ACE2* and *furin* expression in cytotrophoblasts compared to differentiated syncytiotrophoblasts. Intriguingly, the expression of *TMPRSS2* mRNA was significantly increased with exposure to DMSO or hypoxia, compared to the standard condition, which promotes differentiation (Fig. 1C).

### The expression of ACE2 and TMPRSS2 proteins in placental villi and in trophoblasts.

**FIG 2:**
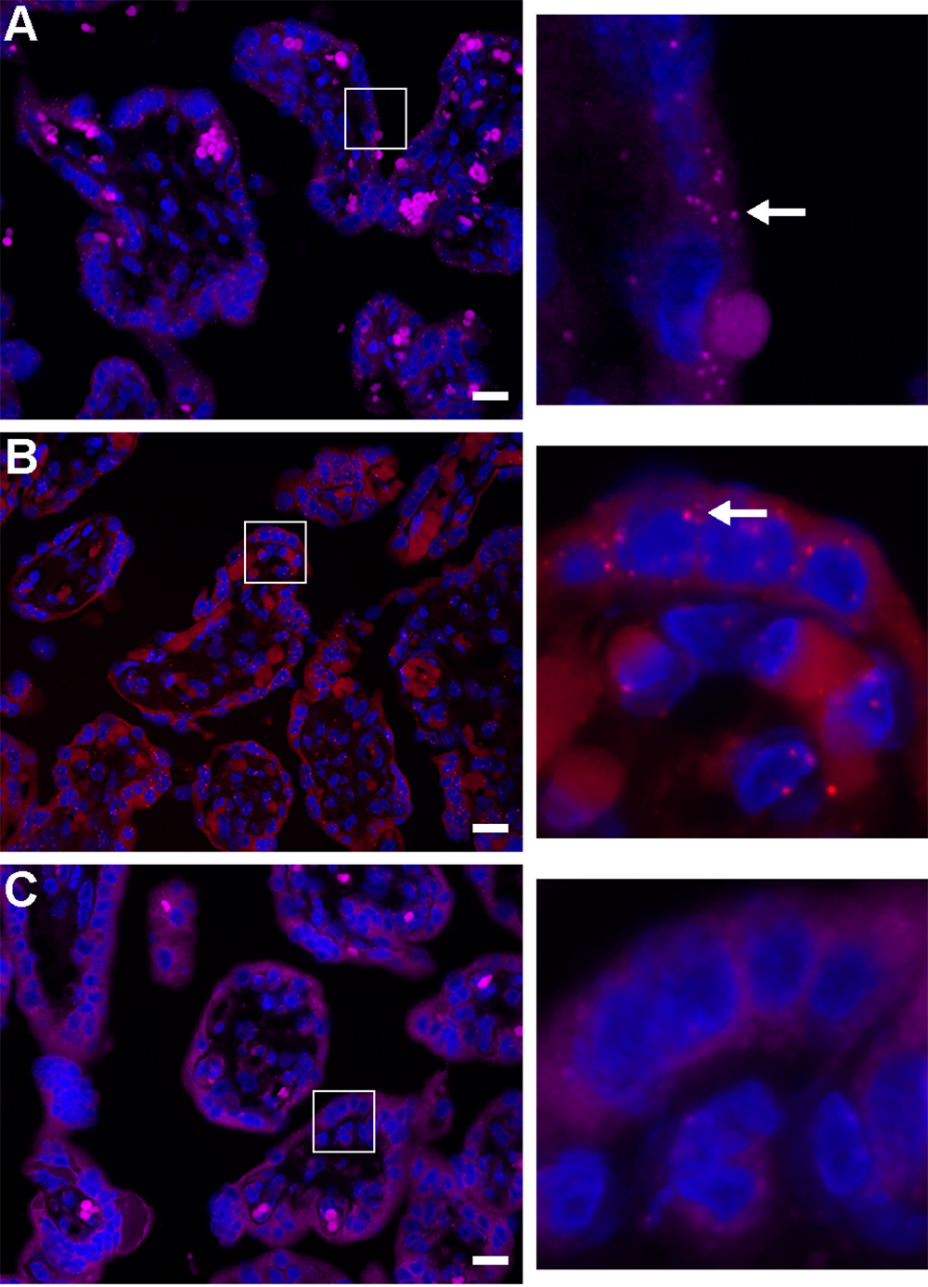
*In situ* hybridization for the expression of ACE2 and TMPRSS2 mRNA in human placental villi. Paraffin-embedded human placental villi sections were processed as detailed in Materials and Methods. RNAscope probes targeting ACE2 (A), TMPRSS2 (B), or control probe (C) were used according to the manufacturer’s instructions. The hybridized probe signal was amplified using the preamplifier reagent followed by the addition of Opal fluorophores. Both ACE2 (A) and TMPRSS2 (B) show notable punctae (white arrow) in the outermost syncytiotrophoblast layer of the placental villi, compared to the negative control (C). The panels on the right are a magnification of the white squares in panels A to C. The panels represent three independent experiments. Bars, 20 μm.

**FIG 3:**
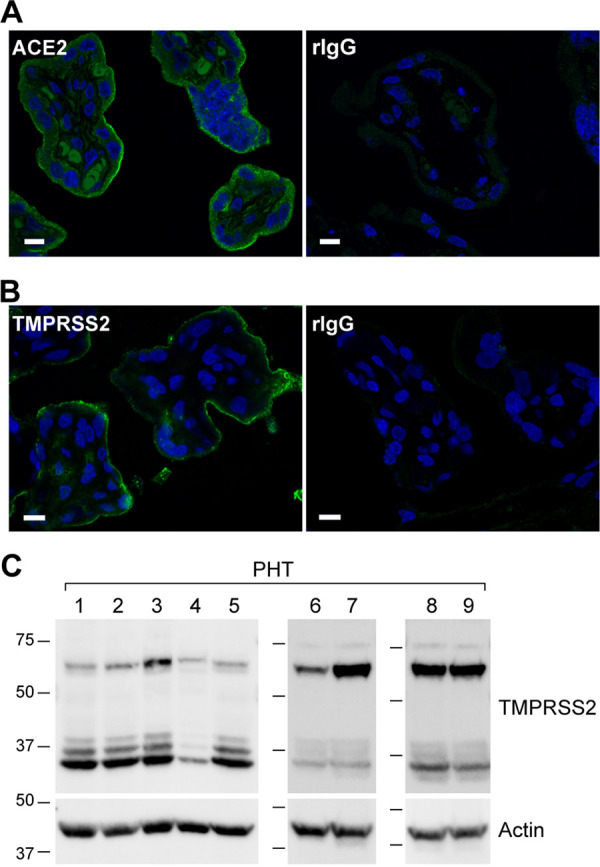
Immunofluorescence localization of ACE2 and TMPRSS2 proteins in the trophoblasts of human placental villi. (A and B) The human placenta villous sections were immunostained with antibodies to ACE2 (A) and TMPRSS2 (B). The concentrations of each pair of specific and control, nonspecific IgG (rIgG), were identical. All image acquisition parameters and intensity scales were identical for each pair of specific and nonspecific antibodies. The panels represent three independent experiments. Bars, 10 μm. (C) Western blot of TMPRSS2 in primary human trophoblasts (PHTs). Whole-cell lysates of PHT cells (60 μg), derived from nine independent placentas, were resolved on SDS-PAGs and transferred to PVDF membranes that were blotted in three independent Western immunoblots using a mouse anti-TMPRSS2 antibody as detailed in Materials and Methods. Note that TMPRSS2 is expressed as a full-length zymogen protein (∼70 kDa) and is processed by proteolytic cleavage (89, 90), producing three activated mature forms with a smaller size (∼37 kDa), derived from two isoforms.

Because of the lower and more selective expression pattern of *ACE2* and *TMPRSS2*, we sought to spatially validate the expression of these two transcripts in trophoblasts. As shown in Fig. 2, we performed *in situ* hybridization using the highly sensitive RNAscope technology. While there were no obvious punctae using the negative-control probe, when using the *ACE2* or *TMPRSS2* probes, we noted prominent punctae in trophoblasts, located at the outermost layer of the placental villi (Fig. 2, arrows in insets). These results confirmed our findings using RT-qPCR and RNA-seq, with regard to trophoblastic expression of *ACE2 and TMPRSS2*. To further corroborate our mRNA expression findings, we examined the expression and location of ACE2 and TMPRSS2 proteins in the placental villi and PHT cells using immunofluorescence labeling. As shown in Fig. 3A and B, ACE2 and TMPRSS2 were significantly expressed in the outer, trophoblast layer at the periphery of the placental villi. Furthermore, we used Western blotting to confirm TMPRSS2 expression in cultured PHT cells (Fig. 3C), providing further support to the expression of SARS-CoV-2 processing proteins in human villous trophoblasts.

**FIG 4:**
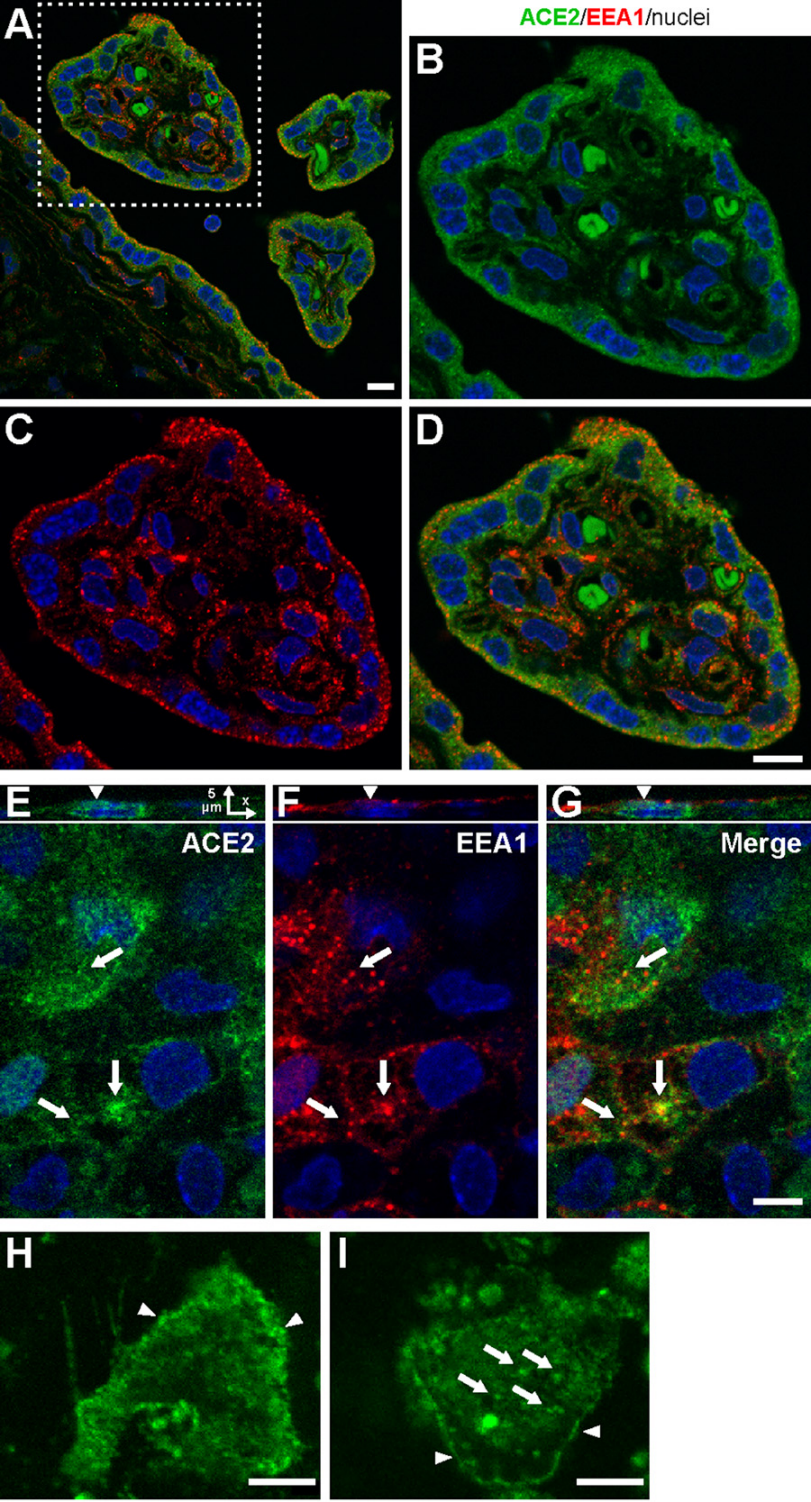
The expression of ACE2 and early endosomal EEA.1 in human placental villi and cultured PHT cells. Placenta crysections were immunolabeled for ACE2 (green) and EEA.1 (red, marker of early/sorting endosomes), and z stacks of *x*-*y* confocal images were acquired. (A) Merged image is shown. (B to D) High-magnification ACE2 and EEA.1 images of a cross section of an individual terminal villus, corresponding to the area marked by the square outlined by a white dashed line in panel A. (E to G) Fixed PHT cells were permeabilized and costained for ACE2 and EEA.1 antibodies. *x*-*z* images in panel E show a single cross section of the three-dimensional (3-D) image. Arrows in panels E to G show examples of ACE2 localization in EEA.1-containing endosomes. Individual *x*-*y* confocal images of 3-D image z stacks are shown. Arrows point at endosomes. (H and I) Representative examples of live-cell images of PHT cells incubated with the Alexa Fluor 488-conjugated ACE2 antibody for 90 min at 37°C. The arrowheads in panel H show ACE2 localization on the plasma membrane, and the arrows in panel I denote the ACE2 cytoplasmic punctae following endocytosis. The panels represent three independent experiments. Bars, 10 μm.

Whereas ACE2 proteins were enriched in the apical membrane of villous trophoblasts, we noted the presence of cytoplasmic punctae in the trophoblasts. This pattern of ACE2 expression prompted us to investigate whether the ACE2 protein undergoes endocytosis in trophoblasts. To address this possibility, we analyzed the colocalization of ACE2 with the canonical early endosome marker EEA.1 in both human villous cryosections and in cultured PHT cells. We found that ACE2- and EEA.1-demarcated endosomes were expressed in villous trophoblasts, with a lower-level expression in the villous core (Fig. 4A to D). Importantly, cytoplasmic ACE2 punctae partially colocalized with EEA.1 in both placental villi (Fig. 4D) and in cultured PHT cells (Fig. 4E to G), suggesting that trophoblastic ACE2 is endocytosed into early endosomes. To assess this possibility, we incubated PHT cells with an Alexa Fluor 488-conjugated ACE2 antibody that recognizes the extracellular domain of ACE2, and we examined the localization of the ACE2-antibody complex in live PHT cells. As shown in Fig. 4H, ACE2 localized to the plasma membrane and intracellular vesicles (Fig. 4I, arrows), thus directly demonstrating endocytosis of ACE2 in trophoblasts. It should be noted that both immunofluorescence and live-cell imaging revealed considerable heterogeneity in the level of ACE2 expression, pattern of localization and endocytosis within the population of PHT cells, likely due to the diverse differentiation state of cells. Overall, our results indicate that the trophoblasts at the outmost layer of the human placental villi express SARS-CoV-2 entry factors, including ACE2, TMPRSS2, and furin, and that these cells are likely capable of ACE2 endocytosis.

### SARS-CoV-2 pseudovirus can enter PHT cells.

**FIG 5:**
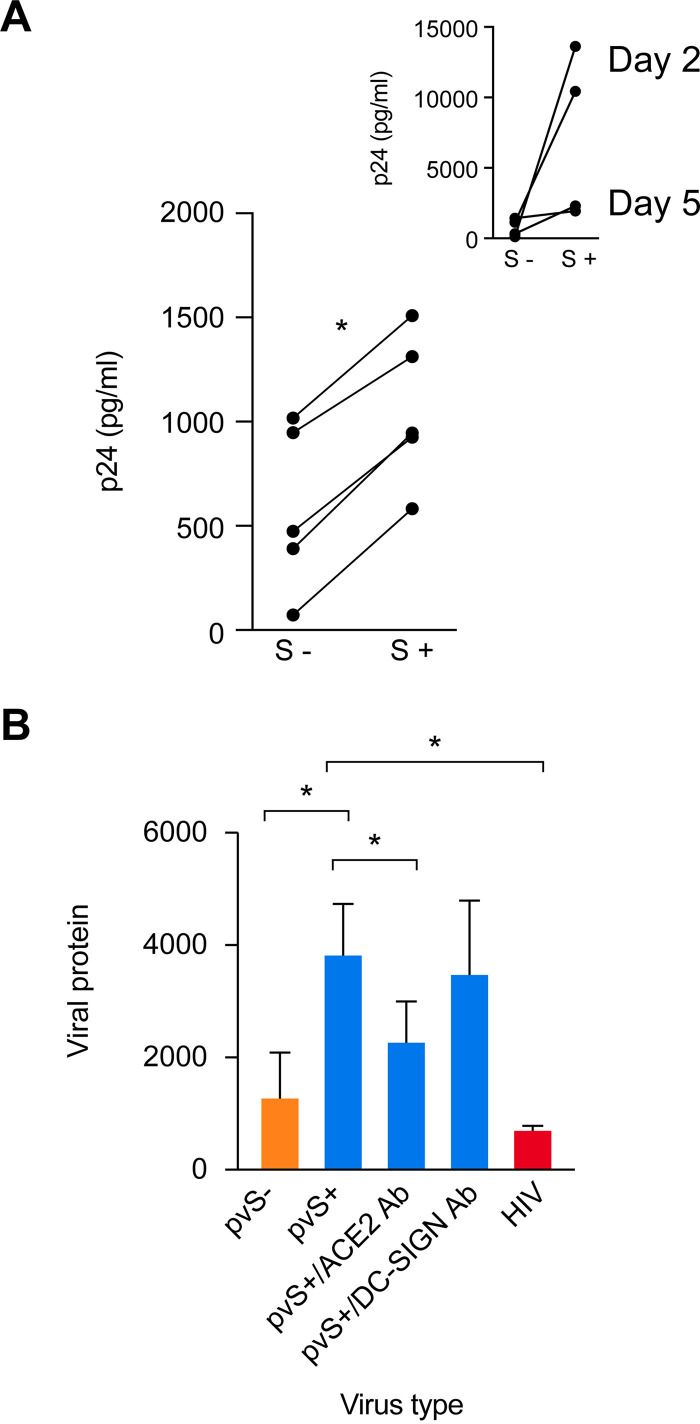
The ACE2-dependent entry of SARS-CoV-2 pseudovirus into cultured PHT cells. (A) PHT (five donors) and 293T-ACE2 cells inoculated with SARS-CoV-2 protein pseudotyped lentiviruses containing SMNE proteins (spike, membrane, nucleocapsid, and envelope) or MNE proteins, as detailed in Materials and Methods. Cells, harvested on day 2 postinoculation with the virus, were washed three times with PBS and trypsinized to remove adherent virus, and the cell pellet was lysed to release intracellular p24. Shown is p24 content (in picograms per milliliter) in cell lysates. *, *P* < 0.01 (paired *t* test). (Inset) 293T cells expressing ACE2, assessed at 2 days or 5 days (*n* = 2 for each) and serving as a positive control. (B) PHT were preincubated or not with 20 μg/ml of anti-ACE-2 or anti-DC-SIGN antibody (Ab) and then inoculated with pvSARS-CoV-2 S+. The PHT cells were also inoculated with pseudovirus lacking S protein (pvS-) or with inactivated HIV-1 as negative controls. Cells were harvested on day 2 postinoculation, and p24 content (in picograms per milliliter) was measured in cell lysates, as detailed in Materials and Methods. *, *P* < 0.05 (ANOVA and Tukey test, *n* = 5).

In light of our findings that the three key SARS-CoV-2 entry factors, ACE2, TMPRSS2, and furin, are expressed in villous trophoblasts, we assessed the susceptibility of PHT cells to entry of replication-deficient lentiviral particles pseudotyped with SARS-CoV-2 viral proteins (pvSARS-CoV-2) membrane (M), nucleocapsid (N), and envelope (E), with (SMNE) or without (MNE) the spike (S) protein. We examined the amount of intracellular lentiviral p24 capsid proteins present in pvSARS-CoV-2 viruses as a measure of cellular viral entry. As expected, we detected robust expression of p24 in HEK293T cells overexpressing ACE2 following 2 days of infection with S-protein-expressing pvSARS-CoV-2, with some decline at day 5 (Fig. 5A, inset), confirming the S-protein dependency of pseudovirus entry. Importantly, PHT cells were infected by pvSARS-CoV-2 SMNE at a significantly higher level than by pvSARS-CoV-2 MNE (Fig. 5A). Overall, the degree of PHT cell infection was markedly lower than that using 293T cells. We confirmed the specificity of pvSARS-CoV-2 S+ entry into PHT cells by preincubating the cells with anti-ACE2 antibody, demonstrating that this significantly reduced viral entry, measured by intracellular p24. This reduction was not observed when we used preincubation with anti-DC-SIGN, which is irrelevant for SARS-CoV-2 entry (Fig. 5B). As expected, PHT cells infected with pvSARS-CoV-2 S- or infected with inactivated HIV with conformationally intact gp160 displayed markedly lower level of viral entry. Overall, these results demonstrate the importance of ACE2 and spike protein for pseudovirus entry into PHT cells. These results are consistent with our expression data, suggesting that human trophoblasts are permissive to pvSARS-CoV-2 entry.

## DISCUSSION

Several lines of evidence support our conclusion that villous placental trophoblasts from term human pregnancies express ACE2, TMPRSS2, and furin. (i) We detected the transcripts for *ACE2*, *TMPRSS2*, and *furin* in term placental trophoblasts by RT-qPCR and RNA-seq. (ii) We used RNAscope to visualize intracellular *ACE2* and *TMPRSS2* mRNA punctae in trophoblasts of the term placental villi. (iii) We identified ACE2 and TMPRSS2 proteins in the term placental villi and in cultured term PHT cells. Whereas furin is expressed in all four cell types we examined, ACE2 and TMPRSS2 are predominantly expressed in the trophoblast layer. Functionally, we demonstrated that cultured PHT cells are permissive to the entry of pvSARS-CoV-2 that express the S protein. Together, our results indicate that the maternal blood-facing villous trophoblasts express the processing proteins that enable SARS-CoV-2 cell entry.

The expression level of ACE2 varies during placental development. While it is expressed in trophoblasts across the various times of gestation surveyed (45, 54, 55, 63, 64), placental *ACE2* transcript levels decline from early pregnancy to term (56, 57). The susceptibility of early gestation trophoblasts to SARS-CoV-2 infection remains unclear, and scant data exist regarding feto-placental transmission of SARS-CoV-2 in early pregnancy (65). Interestingly, we found that PHT cell differentiation, which is enhanced as pregnancy progresses, is associated with higher *ACE2* and *furin* expression, but not TMPRSS2 expression. This might have added to the variable level ACE2 protein expression in trophoblasts. The implications of these findings to feto-placental susceptibility to SARS-CoV-2 infection remain to be defined.

TMPRSS2 is obligatory for cleavage of the viral S2 subunit, thus exposing the fusion peptide that facilitates SARS-CoV-2 cell entry and subsequent viral replication (7, 8). Although others have noted that the expression of TMPRSS2 might be below the detection threshold (45, 49), using several sensitive technologies, we were able to unequivocally document TMPRSS2 mRNA and protein expression in trophoblasts, but not in other villous cell types. Placental expression of furin endopeptidase has previously been demonstrated, where it was shown to be required for proteolytic activation of two fusogenic proteins, syncytin-1 and syncytin-2, which are essential for trophoblast fusion (66–69). Unlike the closely related coronavirus SARS-CoV, the S protein of SARS-CoV-2 has evolved to harbor a proteolytic cleavage site of furin, leading to the production of two subunits, S1 and S2, in SARS-CoV-2 permissive cells (70). Importantly, furin-mediated processing of the S protein is critical for SARS-CoV-2-induced cytopathic effects on host cells, cell-cell fusion-mediated viral spread, and SARS-CoV-2 production (8, 9, 71).

Following the initial interaction of SARS-CoV-2 with its receptor, ACE2, on the plasma membrane (6), SARS-CoV-2 usurps the endocytic pathway, culminating in release of its viral genome for viral propagation and assembly of progeny virions. Blocking endocytosis impairs SARS-CoV-2 replication (70, 72), suggesting that proper spatiotemporal trafficking and sorting of SARS-CoV-2 within the endosomal network is required for viral replication in host cells. In line with this finding, we showed that ACE2 protein was colocalized with early endosomes, suggesting that ACE2 and its interacting SARS-CoV-2 are likely to enter endocytic pathways in human trophoblasts. Notwithstanding this observation, current epidemiological data suggest that the feto-placental unit is largely resistant to SARS-CoV-2 infection. Based on these observations, it is likely that SARS-CoV-2 enters trophoblasts, albeit at a lower level than permissive lung epithelial cells. Thereby, we infer that SARS-CoV-2 replication in the human placenta may be limited by the failure to activate postentry pathways, such as endosomal escape or the lysosomal deacidification pathways (73). In line with this intriguing possibility, a recent report demonstrated that interferon-induced transmembrane proteins (IFITM) family member IFITM3, an endosomal protein, restricts SARS-CoV-2 replication via its amphipathic helix domain, which is intimately connected with the endocytosis pathway (74).

Several studies have associated placental histological lesions with COVID-19 infection during pregnancy. While some of these histopathological patterns, such as intervillositis with macrophage or neutrophil infiltration (46–48), are known to accompany other placental viral infections, other lesions are part of maternal or fetal malperfusion lesions (39, 46, 47, 75–78). Whether or not these lesions are caused by SARS-CoV-2 infection or by some other process that enables SARS-CoV-2 villous entry at the site of damage or are a mere coincidence remains to be established.

Current data support the notion that transplacental fetal transmission of SARS-CoV-2 infection is a rare event (12). Our data imply that placental resistance to SARS-CoV-2 infection cannot be assumed on the basis of deficient expression of the SARS-CoV-2 attachment and processing proteins ACE2 and TMPRSS2, as suggested by Pique-Regi et al. (49), but rather is due to post-viral-entry events. Deeper insight into trophoblastic immune response (79) and endocytic pathways will be essential to deciphering the mechanisms underlying resistance to SARS-CoV-2 infection at the maternal-fetal interface.

## MATERIALS AND METHODS

### Cell culture and reagents.

The institutional review board at the University of Pittsburgh approved all placental procurement, dispersal, and experimental protocols used in these studies. PHT cells were dispersed from term placentas, using a modification of previously published trypsin-DNase-dispase/Percoll protocols (59, 80) and maintained up to 72 h in Dulbecco modified Eagle medium (DMEM; Corning, New York, NY) containing 10% bovine growth serum (BGS; HyClone, Logan, UT) and 1% penicillin-streptomycin (Sigma-Aldrich, St. Louis, MO) at 37°C in a 5% CO_2_ air atmosphere. PPF cells were isolated by means of our routine standard placental cell isolation procedures and further purified using a mouse anti-CD9 antibody (catalog no. BS3022; Bioworld, St. Louis Park, MN) conjugated to magnetic beads. PPF cell purity was validated by flow cytometry using a mouse antivimentin antibody (clone V9) (catalog no. M0725; Dako, Carpinteria, CA). PPF cells were cultured in DMEM with 10% BGS and antibiotics. HBCs were isolated after our initial trypsin and DNase isolation of PHT cells, following the technique detailed by Tang et al. (81). The purity of HBC cells was validated using the selective marker CD163, with negative signal for CD90, expressed by PPF and other nonmacrophage cell types (81). HuPEC cells were isolated using a previously described protocol, with modifications (82). The identity of HuPEC cells was confirmed via flow cytometry, with positive expression of CD31, endoglin (CD105), and melanoma cell adhesion molecule (CD146) and a lack of CD90 and protein tyrosine phosphatase receptor type C (CD45).

### RNA extraction, cDNA synthesis, and real-time quantitative PCR (RT-qPCR) or transcriptome sequencing (RNA-seq).

For mRNA analysis, total RNA was extracted using TRI reagent (Thermo Fisher, Waltham, MA) according to the manufacturer’s protocol. RNA samples were further purified with on-column RNase-free DNase (Qiagen, Germantown, MD). We purchased a FirstChoice Human Total RNA Survey Panel from Thermo Fisher (catalog no. AM6000), which consisted of total RNA from various healthy human tissues, including placenta, heart, intestine, kidney, liver, lung, and testis. Total RNA was reverse transcribed using a HiCapacity cDNA synthesis kit (Thermo Fisher) according to the manufacturer’s protocol. Quantitative PCR was performed using SYBR Select in the ViiA 7 system (Thermo Fisher), and results were presented as threshold cycle (*C_T_*) values, or calculated using the 2^−ΔCT^ method (83) and normalized to the expression of the housekeeping gene glyceraldehyde 3-phosphate dehydrogenase (GAPDH). Note that GAPDH was within 1 *C_T_* value across the human tissues and villous cells. All primer sequences were checked for specificity using BLAST (Basic Local Alignment Search Tool) and are presented in Table S1 in the supplemental material. Dissociation curves were run on all reactions to ensure amplification of a single product with the appropriate melting temperature. Control samples of H_2_O were included in each qPCR experiment.

10.1128/mSphere.00250-21.1 TABLE S1 Primers used in the studies. Download Table S1, PDF file, 0.02 MB. Copyright © 2021 Ouyang et al. 2021 Ouyang et al. https://creativecommons.org/licenses/by/4.0/ This content is distributed under the terms of the Creative Commons Attribution 4.0 International license.

RNA libraries of primary human trophoblasts were constructed by different sequencing facilities and sequenced on an Illumina next-generation instrument. After trimming, the RNA sequencing libraries were aligned to human reference genome GRCh38, using the RNA-seq alignment tool STAR (84). The number of reads per gene was calculated for each RNA-seq library, also using STAR. We then used the negative binomial test implemented in the R package DESeq2 (85) to identify *ACE2*, *TMPRSS2*, and *furin* genes that were differentially expressed either between the standard and hypoxia conditions or between the standard and 1.5% DMSO conditions (see also “Statistical analysis” below).

### *In situ* hybridization of ACE2 and TMPRSS2 using RNAscope technology.

Fluorescence *in situ* hybridization was performed using the RNAscope Fluorescent Multiplex kit (catalog no. 323135; Advanced Cell Diagnostics, Newark, CA) according to the manufacturer’s instructions with minor modifications. Briefly, targeting ACE2 (catalog no. 848151-C3) and TMPRSS2 (catalog no. 470341-C1) probes were purchased from Advanced Cell Diagnostics. Paraffin-embedded sections were deparaffinized twice with xylene, each for 5 min, followed by two 100% ethanol washes for 2 min each. After that, tissues were exposed to hydrogen peroxide for 10 min, heated in kit-provided target retrieval buffer for 15 min at 100°C, and then digested by the provided proteinase plus. After digestion, the probes were added and incubated at 40°C for 2 h. After the probes were rinsed, the signal was amplified using the preamplifier reagent, followed by the addition of Opal fluorophores. The reaction was then blocked, the slides were rinsed and mounted with 4′,6-diamidino-2-phenylindole (DAPI)-Vectashield (Vector labs, Burlingame, CA). Sections were imaged with a Zeiss AxioImager M2 microscope with 40× lens and processed using Zeiss Efficient Navigation microscope software (Carl Zeiss AG, Oberkochen, Germany).

### Immunofluorescence of ACE2 and TMPRSS2 in the human placenta.

Cryosections of placental villi were washed and then processed in 1% hydrogen peroxide for 10 min to inhibit endogenous peroxidase. The tissues were then washed, permeabilized with 0.1% Triton X-100 for 1 h, and preincubated with a blocking buffer containing 10% healthy or normal donkey serum (NDS) (catalog no. D9663; Sigma-Aldrich), 3% bovine serum albumin (BSA) (catalog no. A2153; Sigma-Aldrich), 0.1% Triton X-100 (catalog no. X100-500ML; Sigma-Aldrich) in Tris-buffered saline for 1 h at room temperature (RT), followed by incubation with primary antibodies at 4°C in phosphate-buffered saline (PBS) containing 10% NDS, 0.1% Triton X-100, and 3% BSA for 48 h. The following primary antibodies were used: ACE2 (2 μg/ml) (ab15348; ABCAM, Cambridge, MA), TMPRSS2 (1 μg/ml) (ab109131; ABCAM), and normal rabbit IgG (2 μg/ml) for comparison to ACE2, 1 μg/ml for comparison to TMPRSS2 (sc-2027; Santa Cruz Biotechnology, Dallas, TX). After three washes in PBS, the sections were incubated with the corresponding secondary antibodies for 1 h at RT. Nuclei were stained with Hoechst 33342 (catalog no. 62249; Thermo Fisher). All sections were mounted with ProLong Gold Antifade mounting medium (catalog no. P36930; Thermo Fisher).

PHT cells were fixed with freshly prepared 4% paraformaldehyde for 15 min at RT and permeabilized with 0.1% Triton X-100 in PBS containing 0.2% BSA for 3 min at RT. Immunolabeling was then performed with mouse monoclonal antibody ACE2 (E-11; 2 μg/ml) (sc-390851; Santa Cruz Biotechnology) and rabbit polyclonal antibody to EEA.1 (2 mg/ml) (AB-2900; ABCAM) followed by the corresponding secondary antibodies. The nuclei were stained with Hoechst 33342, and cells were mounted with ProLong Gold as described above. To visualize ACE2 traffic in living cells, PHT cells were incubated with Alexa Fluor 488-conjugated ACE2 antibody (4 μg/ml) (sc-390851; Santa Cruz Biotechnology) for 90 min at 37°C, and live-cell imaging was performed at RT.

To obtain high-resolution three-dimensional images of placenta slices and PHT cells, a z stack of confocal images was acquired using a spinning-disk confocal system based on a Zeiss Axio Observer Z1 inverted fluorescence microscope, equipped with a 63× Plan Apo PH, 1.4-numerical-aperture (NA) objective, computer-controlled spherical aberration correction unit, Yokogawa CSU-W1, Vector photomanipulation module, Photometrics Evolve (used for imaging PHT cells), and Hamamatsu Orca-Flash4.0 complementary metal oxide semiconductor (CMOS) (used for imaging of placenta sections) cameras, environmental chamber, piezo stage controller and lasers (405, 445, 488, 515, 561, and 640 nm), all controlled by SlideBook 6 software (Intelligent Imaging Innovations, Denver, CO). Typically, 20 to 30 serial two-dimensional confocal images were recorded at 400-nm intervals. Colocalization of ACE2 with EEA.1 was determined by identifying clear overlapping structures that could be followed in multiple *z* planes.

### Western immunoblotting.

Cells were lysed on ice for 30 min in lysis buffer (20 mM HEPES [pH 7.5], 150 mM NaCl, 1% Triton X-100) supplemented with protease inhibitor cocktail mini EDTA-free and PhoSTOP (Roche, Mannheim, Germany) and centrifuged at 16,000 × *g* at 4°C for 10 min to remove cell debris. Lysate concentrations were determined with Pierce bicinchoninic acid (BCA) protein assay kit (Thermo Fisher) using a Versa Max microplate reader (Molecular Devices, San Jose, CA). Protein samples (60 μg) were resolved on a sodium dodecyl sulfate-polyacrylamide gel (SDS-PAG), and transferred onto 0.2-μm polyvinylidene difluoride (PVDF) membrane (Bio-Rad, Hercules, CA), using standard procedures. The PVDF membranes were immunoblotted with a mouse monoclonal anti-actin antibody (MAB1501; Sigma) (diluted 1:20,000) and a mouse anti-TMPRSS2 antibody (sc-515727; Santa Cruz Biotechnology) (diluted 1:500). The blots were processed for chemiluminescence using a WesternBright Sirius kit (catalog no. K-12043-D20; Advansta, San Jose, CA) and imaged with the ChemiDoc system (Bio-Rad).

### Pseudotyped virus entry of primary human trophoblasts.

PHT cells and 293T-ACE2 (Integral Molecular, Philadelphia, PA) were seeded in 12-well plates at a density of 1.3 × 10^6^ cells/well for PHT (five donors) and 3 × 10^5^ cells/well for 293T-ACE2. Cells were infected with pvSARS-CoV-2 lentiviruses (100 ng/ml based on the p24 content) (Virongy, Manassas, VA) containing SMNE proteins (spike, membrane, nucleocapsid, and envelope), MNE proteins, S protein only, or no SARS-CoV-2 proteins, as well as aldrithiol-inactivated HIV-1 (86) as a negative control. For antibody inhibition experiments, PHT cells were preexposed to 20 μg/ml anti-ACE2 (catalog no. AF933; R&D Systems, Minneapolis, MN ) or anti-DC-SIGN (R&D catalog no. MAB161) for 30 min at 37°C before inoculation with the viruses (1, 7). Cells were harvested at day 2 and day 5 postinfection, and the cells were washed three times with 2 ml of PBS followed by 0.25% trypsin-EDTA for 10 min to release the cells from plastic and to remove any virus adhering to them. The cells were centrifuged at 400 × *g* for 5 min to pellet cells, which was lysed with 1% Triton X-100 for 15 min at 37°C and centrifuged at 2,000 × *g* for 5 min. The lysate supernatant was collected, and p24 was measured in the cell lysate using a cytometric bead assay (87). Values were adjusted to normalize for volume differences (1 ml for each fraction).

### Statistical analysis.

The main statistical analyses were performed with Prism software (GraphPad, San Diego, CA). Statistical significance for multiple comparison was calculated by one-way analysis of variance (ANOVA) and Tukey *post hoc* test or by two-tailed paired or unpaired *t* test, where appropriate. Significance was determined as *P* < 0.05. Values are presented as means ± standard deviations (SD), derived from at least three independent experiments, as indicated in each figure legend. For statistical analysis of the RNA-seq-based expression of *ACE2*, *TMPRSS2*, and *furin* among the various experimental groups, the *P* values of differential expression tests were adjusted using Benjamini and Hochberg’s method (88) to control the false discovery rate. In the plots, the expression of the genes was first normalized by the median of ratio method implemented in DESeq2 (85) and then log_2_ transformed.

### Data availability.

RNA-seq data have been deposited with the NCBI BioProject, accession numbers PRJNA674312, PRJNA674329, and PRJNA674366.
