# Supplementary material for: Term Human Placental Trophoblasts Express SARS-CoV-2 Entry Factors ACE2, TMPRSS2, and Furin
Source: mSphere. 2021 Apr 14;6(2):e00250-21. doi: 10.1128/mSphere.00250-21 (PMC8546705; doi:10.1128/mSphere.00250-21)
Supplement: TABLE S1 [file msphere.00250-21-st001.pdf]

SUPPLEMENTAL MATERIAL: Term human placental trophoblasts express SARS-CoV-2 entry factors ACE2, TMPRSS2, and furin

Yingshi Ouyang, Tarique Bagalkot, Wendy Fitzgerald, Elena Sadovsky, Tianjiao Chu, Ana Martínez-Marchal, Miguel Brieño-Enríquez, Emily J. Su, Leonid Margolis, Alexander Sorkin, Yoel Sadovsky

Table S1. Primers used in the studies.

Name	Direction	ID	Sequence
ACE2	Forward	NM_021804	CGAAGCCGAAGACCTGTTCTA
	Reverse		GGGCAAGTGTGGACTGTTCC
Furin	Forward	NM_002569	CCTGGTTGCTATGGGTGGTAG
	Reverse		AAGTGGTAATAGTCCCCGAAGA
TMPRSS2	Forward	NM_001135099	GTCCCCACTGTCTACGAGGT
	Reverse		CAGACGACGGGGTTGGAAG
GAPDH	Forward	NM_002046.7	GAAGGTCGGAGTCAACGGATTT
	Reverse		GAATTTGCCATGGGTGGAAT
